# 

**Published:** 1879-05

**Authors:** 


### Table of Contents.

### ORIGINAL COMMUNICATIONS.

Case of Retained Menstrual Matter from Occluded Os. *By E. Griffin, M.D., Atlanta, Ga* ..... 65

### REPORTS OF SOCIETIES.

Atlanta Academy of Medicine ..... 67

### EDITORIAL.

Paper on Quarantine ..... 74
Convention of American Medical Colleges ..... 77
American Medical College Association ..... 83
National Board of Health ..... 90
American Medical Association ..... 109
Meteorological Report for April ..... 122
An Agreeable Surprise ..... 123
In Memoriam ..... 123

### BIBLIOGRAPHICAL.

A Manual of Examination of the Eyes ..... 123
Health, and How to Promote It ..... 124
Health Primers ..... 124
Atlas of Skin Diseases ..... 125
Pamphlets Received ..... 125

### EXCERPTA.

Rare Form of Intestinal Obstruction—Operation—Cure ..... 126
Abscess of the Tonsils ..... 127
The Tapeworm ..... 128

